# Liver cirrhosis in metabolic dysfunction-associated steatohepatitis

**DOI:** 10.1093/gastro/goaf037

**Published:** 2025-05-02

**Authors:** Donghyun Ko, Do Han Kim, Pojsakorn Danpanichkul, Masahito Nakano, Chitchai Rattananukrom, Karn Wijarnpreecha, Cheng Han Ng, Mark D Muthiah

**Affiliations:** Department of Medicine, Bridgeport Hospital Yale New Haven Health, Bridgeport, CT, USA; Department of Medicine, Mount Sinai Morningside and West, Icahn School of Medicine at Mount Sinai, New York, NY, USA; Department of Medicine, Texas Tech University Health Sciences Center, Lubbock, TX, USA; Division of Gastroenterology, Department of Medicine, Kurume University School of Medicine, Kurume, Fukuoka, Japan; Division of Gastroenterology and Hepatology, Department of Medicine, Faculty of Medicine, Srinagarind Hospital, Khon Kaen University, Khon Kaen, Thailand; Department of Medicine, Division of Gastroenterology and Hepatology, University of Arizona College of Medicine, Phoenix, AZ, USA; Department of Internal Medicine, Division of Gastroenterology and Hepatology, Banner University Medical Center, Phoenix, AZ, USA; BIO5 Institute, University of Arizona College of Medicine-Phoenix, Phoenix, AZ, USA; Division of Gastroenterology, Department of Medicine, Kurume University School of Medicine, Kurume, Fukuoka, Japan; Graduate School of Medicine, Yokohama City University, Yokohama, Japan; Division of Gastroenterology and Hepatology, Department of Medicine, National University Hospital, Singapore, Singapore; Division of Gastroenterology and Hepatology, Department of Medicine, National University Hospital, Singapore, Singapore; Yong Loo Lin School of Medicine, National University of Singapore, Singapore, Singapore; National University Centre for Organ Transplantation, National University Health System, Singapore, Singapore

**Keywords:** MASH, cirrhosis, progression, randomized clinical trial, observational study, outcomes

## Abstract

In the present narrative review, we have summarized the current evidence on the natural progression of metabolic dysfunction-associated steatohepatitis (MASH) cirrhosis observed through the placebo arm in clinical trials and observational studies. The outcomes scrutinized throughout our review were histology-related changes, non-invasive fibrosis markers, indicators of decompensation, end-stage hepatic complications, and mortality reported during the different clinical trials. Given the short duration of clinical trials, observational studies were included to obtain better insight into the long-term progression and prognosis of MASH cirrhosis. Lastly, new updates about MASH cirrhosis treatments were listed, and the results of these randomized clinical trials were described to enhance our understanding of our current standing in the treatment of MASH cirrhosis.

## Introduction

Metabolic dysfunction-associated steatotic liver disease (MASLD) and metabolic dysfunction-associated steatohepatitis (MASH) have been the terms to better describe non-alcoholic fatty liver disease (NAFLD) and non-alcoholic steatohepatitis (NASH) since June 2023 [1]. With these changes, there were concerns that the change in terminology might have significantly impacted in the outcome in this set of patients. Pennisi *et al.* [2] have extensively described that MASLD patients had significantly higher rates of overall mortality compared to NAFLD; however, another study by Younossi *et al.* [3] discussed that the clinical profiles are identical, allowing the interchangeable use of both terminologies. There is no consensus on whether studies should unify these terms, but in our review, we will use MASH to describe NASH.

Currently, MASLD represents the most common chronic liver disease worldwide, with a prevalence of 38% of all adults, and it is projected to increase to over 55% by 2040 [4]. The incidence of MASLD is increasing with an estimated 4,613 new cases per 100,000 person-years with significantly higher incidence rates in males and overweight/obese individuals [5]. Although MASLD is known to have less evident risk of progression to advanced fibrosis, MASH is generally considered to have a significant risk of progressing into advanced fibrosis and liver-related mortality [6, 7]. Singh *et al.* [6] have estimated that the annual fibrosis progression rate in patients with MASLD vs MASH was 0.07 stages (95% confidence interval [CI], 0.02–0.11 stages) vs 0.14 stages (95% CI, 0.07–0.21 stages), respectively, which corresponds to an average progression of 1 stage over 14.3 vs 7.1 years, respectively. In a study led by Argo *et al.* [8], the mean rate of progression in patients with MASH was 0.03 stages per year.

**Figure 1.**
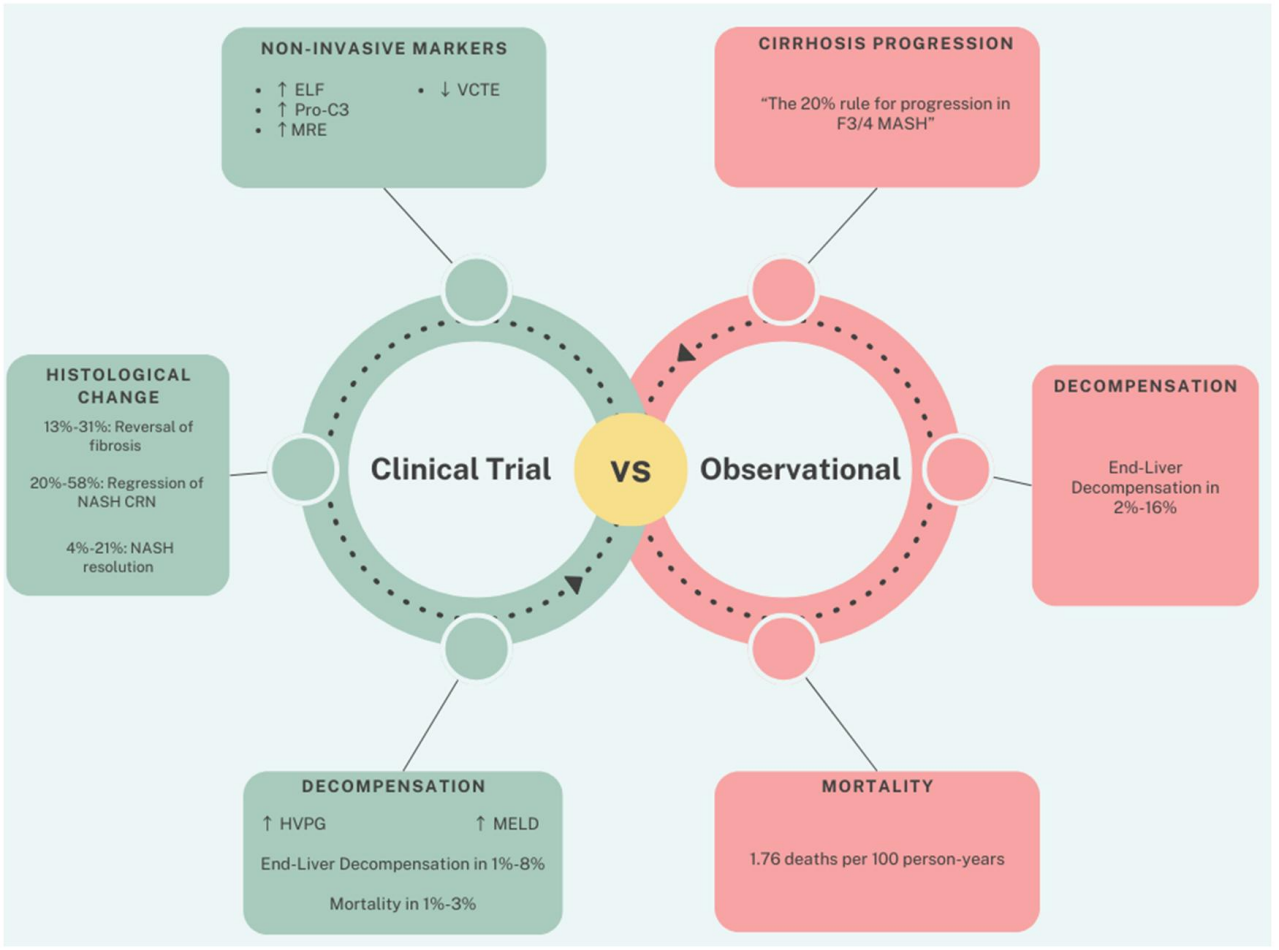
Summary of progression of MASH cirrhosis in clinical trial vs observational studies.

Ng *et al.* [9] demonstrated that all-cause mortality in patients with MASH cirrhosis was 0.3%, 13.0%, 20.6%, 33.3%, and 41.5% at 1, 3, 5, 8, and 10 years. Much of our current understanding is driven by observational studies, which have provided novel insight into the natural history of liver disease in patients with MASLD and MASH [10–12]. However, there have been recent endeavors by multiple pharmaceutical companies to develop treatments specifically for MASH cirrhosis. Through these new randomized clinical trials (RCTs) published over the last few years, detailed data are available to describe the progression of MASH cirrhosis. In this review, we have collected observational studies and RCTs to explain the different outcomes of MASH cirrhosis. Additionally, we have included recent updates on treatments in development, specifically in cirrhosis developed from MASH. The main findings are summarized in Figure 1.

## Natural history of MASH cirrhosis: evidence from RCTs

### Histology-related measures

RCTs provide a unique and controlled environment for studying MASH by observing disease progression in the placebo arm. A meta-analysis by Ng *et al.* [13] found that the pooled estimate of MASH resolution and 2-point NAFLD activity score (NAS) reduction without worsening of fibrosis was 11.65% (95% CI, 7.98–16.71) and 21.11% (95% CI, 17.24–25.57); furthermore, the rate of ≥1 stage reduction and progression of fibrosis was 18.82% (95% CI, 15.65–22.47) and 22.74% (95% CI, 19.63–26.17), respectively, among the entire population of MASH recruited for clinical trial. To date, 10 RCTs have been conducted specifically in MASH cirrhosis, with the duration of studies ranging from 26 to 96 weeks (Table 1) [14–23]. Using histological endpoints, reversal of fibrosis was defined as ≥ 1 stage improvement in fibrosis without worsening of MASH, which occurred from 13% to 31% of patients. In the Efruxifermin trial, a 26-week study led by Harrison *et al.* [18] found that none of the MASH cirrhosis patients under placebo had reversal of fibrosis. At week 48, Loomba *et al.* [22] reported reversal of fibrosis in 29% of MASH cirrhosis patients under placebo. Regression of NASH Clinical Research Network NAS was defined by ≥2-point improvement in NAS, which occurred in 20% to 58% of patients. At week 26, 20% of MASH cirrhosis patients under placebo had regression of NAS [18]. At week 48, 44% of MASH cirrhosis patients under placebo had regression of NAS. Lastly, at week 96, 22% of MASH cirrhosis patients under placebo had regression of NAS (Table 2) [20]. As for isolated MASH resolution, which occurred in 4% to 21%, Loomba *et al.* [22] found that 21% of patients under placebo had MASH resolution at week 48. Conversely, in the Simtuzumab trial led by Harrison *et al.* [20], only 4% of patients under placebo had MASH resolution at week 96.

**Table 1.** Improvement of fibrosis throughout the included randomized clinical trials

Author	Year	Duration (weeks)	≥1 stage improvement in fibrosis NASH CRN (%)	≥1 stage improvement in Ishak fibrosis stage (%)	≥1 stage improvement in fibrosis without worsening of MASH (%)	MASH resolution (%)
Harrison *et al.* [18]	2022	26	N/A	N/A	0/5 (0)	0/5 (0)
Loomba *et al.* [22]	2023	48	8/24 (33)	N/A	7/24 (29)	5/24 (21)
Harrison *et al.* [19]	2020	48	27/172 (16)	N/A	22/172 (16)	7/172 (4)
Loomba *et al.* [21]	2021	48	N/A	N/A	3/21 (14)	0/21 (0)
Rinella *et al.* [23]	2024	48	8/76 (15)	N/A	7/56 (13)	N/A
Abdelmalek *et al.* [14]	2024	48	13/39 (33)	14/39 (36)	12/39 (31)	0/39 (0)
Chalasani *et al.* [15]	2020	52	N/A	14/54 (26)	N/A	N/A
Harrison *et al.* [20]	2018	96	6/75 (8)	11/75 (15)	N/A	3/55 (6)

NASH = non-alcoholic steatohepatitis, CRN = Clinical Research Network, MASH = metabolic dysfunction-associated steatohepatitis.

**Table 2.** Regression of NASH CRN: NAS throughout the included randomized clinical trials

Author	Year	Duration (weeks)	≥2-point improvement in NAS	NAS: Steatosis improvement	^a^NAS: Lobular inflammation improvement	NAS: Hepatocellular ballooning improvement
Harrison *et al.* [18]	2022	26	1/5 (20%)	N/A	N/A	N/A
Loomba *et al.* [22]	2023	48	14/24 (58%)	8/24 (33%)	9/24 (38%)	8/24 (33%)
Abdelmalek *et al.* [14]	2024	48	14/39 (36%)	4/39 (10%)	8/39 (21%)	5/39 (13%)
Chalasani *et al.* [15]	2020	52	N/A	0.2 (0.6) mean (SD)	0.1 (0.8) mean (SD)	0.3 (0.7) mean (SD)
Harrison *et al.* [20]	2018	96	16/72 (22%)	N/A	N/A	N/A

a Lobular inflammation: lobular inflammation grade 3 improvement; hepatocellular ballooning improvement: hepatocellular ballooning grade 2 improvement.

NAS = NAFLD activity score, SD = standard deviation.

### Non-invasive markers of fibrosis

Non-invasive fibrosis measurements commonly used in clinical trials included enhanced liver fibrosis (ELF), vibration-controlled transient elastography (VCTE), N-terminal propeptide of type III collagen (PRO-C3), and magnetic resonance elastography (MRE). Overall, MASH cirrhosis patients on placebo had increased ELF of 0.4 (0.0–0.6) median absolute change from baseline (CFB [interquartile range—IQR]) at week 26 [18], 0.3 (0.1) least-square mean change (SE) [23] at week 48, and 0.37 (0.63) mean change (SD) [15] at week 52. Similarly, PRO-C3 had 12.7 ng/mL (6.2) least-square mean change (SE) compared to baseline at week 48 [23]. MRE had increased from baseline at week 48 with a change from 6.08 kPa (1.99) to 6.22 kPa (2.87) mean (SD) [22]. Lastly, VCTE had decreased at week 48 with a change from 20.00 kPa (14.4–26.7) to 19.30 kPa (13.8–26.7) median (IQR) (Table 3) [19].

**Table 3.** Changes in non-invasive markers of fibrosis throughout the included randomized clinical trials

Author	Year	Duration (weeks)	ELF change	Pro C3	VCTE	MRE
Harrison *et al.* [18]	2022	26	0.4 (0.0, 0.6) median absolute CFB (IQR)	−3.4 (−6.9, 0.2) LS mean absolute CFB (IQR)	N/A	N/A
Garcia-Tsao *et al.* [17]	2020	48	N/A	N/A	−0.3 (14.3) mean (SD)	N/A
Loomba *et al.* [22]	2023	48	−0.13 LS mean change	N/A	N/A	6.08 (1.99) to 6.22 (2.87) mean (SD)
Harrison *et al.* [19]	2020	48	10.67 (10.05–11.16) to 10.66 (10.14–11.26) median (IQR)	N/A	20.00 (14.4–26.7) to 19.30 (13.8–26.7) median (IQR)	N/A
Rinella *et al.* [23]	2024	48	0.3 (0.1) LS mean change (SE)	12.7 ng/mL (6.2) LS mean change (SE)	N/A	N/A
Chalasani *et al.* [15]	2020	52	0.37 (0.63) mean change (SD)	N/A	N/A	N/A
Harrison *et al.* [20]	2018	96	10.80 (10.05–11.50) to 10.97 (9.89–11.59) median (IQR)	N/A	N/A	N/A

ELF = enhanced liver fibrosis, Pro C3 = N-terminal propeptide of type III collagen, VCTE = vibration-controlled transient elastography, MRE = magnetic resonance enterography, CFB = change from baseline, LS = least-square, IQR = interquartile range, SD = standard deviation, SE = standard error.

### Indicators of decompensation

In the Emricasan, Belapectin, and Simtuzumab trials, hepatic vein pressure gradient (HVPG) measurements were performed during the screening period and at the corresponding weeks at the end of the trial. HVPG measurements were made by percutaneous catheterization of the hepatic vein using balloon-tipped catheters and interpreted in mean change in mmHg [24]. Study has shown that HVPG (per mmHg; adjusted subdistribution hazard ratio [aSHR] 1.12, 95% CI 1.07–1.18) was a key measure associated with the development of decompensation [25]. At week 24, patients under placebo had a mean (SD) change of +0.33 mmHg (2.3) [17]. At week 52, there was a mean (SE) change of +0.40 mmHg (0.57) [15]. Lastly, in week 96, there was a mean (SD) change of −0.1 mmHg (4.01) [20]. The model for end-stage liver disease (MELD) is a tool to predict mortality risk in cirrhotic patients. It has been validated as a predictor of short- and medium-term survival and is associated with decreased residual liver function [26]. At week 24, 8% of patients under placebo had progression to MELD ≥ 15, and at week 48, 4% of patients under placebo had progression to MELD ≥ 15 [17]. In terms of the average increase in MELD score at week 48, Frenette *et al.* [16] reported an average increase of 1.8 (2.31) mean (SD) in MELD score, while Loomba *et al.* [22] reported an average increase of 0.1 (3.1) mean (SD) (Table 4). Generally, the trend of MELD score in MASH cirrhosis is to increase as the disease progresses, reflecting worsening liver function and higher mortality risk.

**Table 4.** Changes in HVPG and MELD throughout the included randomized clinical trials

Author	Year	Duration (weeks)	HVPG	MELD	MELD score ≥ 15
Garcia-Tsao *et al.* [17]	2020	48	0.33 mmHg (2.3) mean CFB (SD)	0.4 (2.2) mean (SD)	4/53 (7.5%)
Loomba *et al.* [22]	2023	48	N/A	0.1 (3.1) mean (SD)	N/A
Harrison *et al.* [19]	2020	48	N/A	7 (6–8) to 7 (6–8) median (IQR)	N/A
Frenette *et al.* [16]	2021	48	N/A	1.8 (2.31) mean (SD)	N/A
Chalasani *et al.* [15]	2020	52	0.40 mmHg (0.57) mean change (SE)	N/A	2/54 (3.7%)
Harrison *et al.* [20]	2018	96	−0.1 mmHg (4.01) mean change (SD)	7 (6–8) to 7 (6–8) median (IQR)	3/85 (4%)

HVPG = hepatic vein gradient pressure, MELD = model for end stage liver disease, ALT = alanine transaminase, AST = aspartate aminotransferase, CFB = change from baseline, LS = least-square, IQR = interquartile range, SD = standard deviation, SE = standard error.

### End-stage hepatic complications

End-stage hepatic complications in cirrhosis were defined as spontaneous bacterial peritonitis, ascites, hepatic encephalopathy, newly diagnosed esophageal varices, or variceal bleeding throughout the trials. The general rate of complications ranged from 1% to 8% among patients on placebo, and the most frequent complication observed was ascites. At week 48, Garcia-Tsao *et al.* [17] reported none with variceal bleeding, 7.3% with newly diagnosed ascites, and 1.8% with hepatic encephalopathy. Conversely, Frenette *et al.* [16] reported 1.4% with variceal bleeding, 1.4% with newly diagnosed ascites, 1.4% with hepatic encephalopathy, and 1.4% with spontaneous bacterial peritonitis. At week 52, Chalasani *et al.* [15] reported none with variceal bleeding, 1.8% with newly diagnosed ascites, 1.8% with hepatic encephalopathy, and 1.8% with spontaneous bacterial peritonitis. Lastly, at week 96, Harrison *et al.* [20] reported none with variceal bleeding, 7% with newly diagnosed ascites, and 2% with hepatic encephalopathy.

### Liver-related mortality

Lastly, liver-related mortality was rarely observed during the duration of the trials. Throughout the trials included in our review, the general rate ranged between 1% and 2.9% among patients on placebo. At week 48, Frenette *et al.* [16] reported that 2.9% of patients under placebo had suffered liver-related death. Moreover, at week 96, Harrison *et al.* [20] reported that 1% of patients under placebo had suffered liver-related death (Table 5). The incidence of liver-related mortality was not frequently observed, and the speculation is that the timeframe of the trials is too short to observe this outcome, especially in trials with baseline compensated MASH cirrhosis.

**Table 5.** Incidence of end-organ complications throughout the included randomized clinical trials

Author	Year	Duration (weeks)	VB	Ascites	HE	SBP	Liver-related mortality
Garcia-Tsao *et al.* [17]	2020	48	0/55	4/55 (7.3%)	1/55 (1.8%)	N/A	N/A
Harrison *et al.* [19]	2020	48	0/172	0/172	1/172 (0.58%)	N/A	0/172
Frenette *et al.* [16]	2021	48	1/70 (1.4%)	1/70 (1.4%)	1/70 (1.4%)	1/70 (1.4%)	2/70 (2.9%)
Rinella *et al.* [23]	2024	48	0/56	0/56	0/56	N/A	N/A
Abdelmalek *et al.* [14]	2024	48	3/39	0/39	N/A	N/A	N/A
Chalasani *et al.* [15]	2020	52	0/54	1/54 (1.8%)	1/54 (1.8%)	1/54 (1.8%)	0/54
Harrison *et al.* [20]	2018	96	0/85	6/85 (7%)	2/85 (2%)	N/A	1/85 (1%)

VB = variceal bleeding, HE = hepatic encephalopathy, SBP = spontaneous bacterial peritonitis.

## Natural history of MASH cirrhosis from observational studies

The understanding of MASH cirrhosis and its progression into decompensated cirrhosis remains unclear. The progression of MASH cirrhosis has yet to be well established throughout the studies. There was an interesting comment by Loomba *et al.* [27], which is the “The 20% rule for progression in F3/4 NASH,” stating that approximately 20% of MASH cirrhosis will develop decompensated cirrhosis in 2 years. However, another study by Allen *et al.* [28] states that the progression of MASH cirrhosis is dynamic, and often, there are limitations to linking longitudinal changes in histologic parameters or non-invasive markers to main liver outcomes with true impact on the real world. Although the progression velocity should be individualized in the clinical scenario, we have included studies that might glimpse the prospects for progressing MASH cirrhosis.

## Timeframe: compensated MASH cirrhosis to decompensation

The timeframe of MASH cirrhosis progressing into decompensated cirrhosis has been a major center of attention in the development of MASH cirrhosis treatment, as its understanding can aid in establishing better clinical outcomes in future trials. A study led by Allen *et al.* [28] found that the progression risk and time spent in each MASH cirrhosis state were explored during a median follow-up of 2.3 years. Overall, patients with compensated MASH cirrhosis spent a mean time of 4.1 years in this state, and these patients had a 4-year cumulative incidence of progression into decompensation, hepatocellular carcinoma, or liver transplant of 32.7% (95% CI, 24.44%–43.83%). For patients with one decompensation, they spent a mean of 2.3 years in this state, and the 2-year cumulative incidence of progression toward more than one decompensation, hepatocellular carcinoma, or liver transplant was 48.1% (95% CI, 27.15%–59.32%). Lastly, patients with more than one decompensation spent a mean of 2.1 years in this state, and the 2-year cumulative incidence of death was 45.8% (95% CI, 35.62%–58.79%).

## End-stage hepatic complications

The heterogeneity in the definition of *End-Organ Hepatic Complications* in cohort studies poses a challenge in the generalization of the results. For example, in a prospective study of outcomes in adults with MASLD by Sanyal *et al.* [29], the end-organ complications only included new onsets of clinically obvious ascites, overt encephalopathy, or variceal hemorrhage. Meanwhile, Sebastiani *et al.* [30] defined end-organ hepatic complications as hepatocellular carcinoma, ascites, spontaneous bacterial peritonitis, hepatic encephalopathy, de novo varices, or significant worsening of varices. Summarized, the rate of these events was 2%, as reported by Sanyal *et al.* [29], and 16.2%, as reported by Sebastiani *et al.* [30]. By large, a study by Fujii *et al.* [31] reported that the incidence of liver-related events, which was defined as variceal bleeding, ascites, and hepatic encephalopathy, per 1,000 person-years among F4 fibrosis was 90.1 (95% CI, 40.1–160); for hepatocellular carcinoma was 16.9 (95% CI, 4.23–67.7). Compared to advanced fibrosis (F3), Vilar-Gomez *et al.* [32] showed that patients with F4 were observed to have more hepatic decompensation (44%; 95% CI, 32%–60% vs 6%, 95% CI, 2%–13%) and HCC (17%; 95% CI, 8%–31% vs 2.3%, 95% CI, 1%–12%).

## Mortality

It is well described that F4 fibrosis is highly associated with overall mortality, liver-related mortality, and cardiovascular mortality. In terms of overall mortality, a prospective study led by Sanyal *et al.* [29] using the NASH Clinical Research Network cohort reported 1.76 deaths per 100 person-years during a median duration of follow-up of 4 years among patients with F4. Additionally, Fujii *et al.* [31] show that the incidence per 1,000 person-years of overall mortality in patients with F4 fibrosis is 24.2 (95% CI, 7.82–75.1). Conversely, Simon *et al.* [33] reported an incidence per 1,000 person-years of 70.5 (95% CI, 63.9–77.5). In terms of risk of mortality in F4 fibrosis, Angulo *et al.* [34] report that F4 fibrosis is significantly associated with death or liver transplantation (hazard ratio [HR] 10.9, 95% CI 6.06–19.62) during a median follow-up of 12.6 years compared to F0. This was similarly reflected in a meta-analysis by Ng *et al.* [13], where the risk of all-cause mortality was highest among all stage of fibrosis in F4 (HR 3.66, 95% CI 2.65–5.05) when compared to F0. A liver transplant waitlist study also shows a good insight into the expected mortality in MASH cirrhosis patients. Lim *et al.* [35] report an increased overall 90-day (HR 1.15, 95% CI 1.07–1.24) and 1-year mortality (HR 1.25, 95% CI 1.16–1.34) in MASH cirrhosis when compared to non-MASH cirrhosis. Another cause of mortality frequently associated with MASH cirrhosis was cardiovascular-related mortality. Grossly the incidence per 1,000 person-years of cardiovascular-related mortality was 15.1 (95% CI, 12.2–18.5) [33].

Specifically for liver-related mortality, similar results were observed by Fujii *et al.* [31] showing an identical incidence per 1,000 person-years as to overall mortality, 24.2 (95% CI, 7.82–75.1) and Simon *et al.* [33] who reported an incidence per 1,000 person-years of 22.4 (CI 95%, 18.8–26.5). Deaths due to hepatocellular carcinoma were analyzed separately in some studies. Overall, hepatocellular carcinoma-related mortality had an incidence per 1,000 person-years of 5.7 (95% CI, 4.0–7.8) [33]. As a general rule, the risk of severe liver disease increased per stage of fibrosis (hazard ratio ranging from 1.9 in F0 to 104.9 in F4) and the lower end of the 95% CI for the 10th percentile of time to development of severe liver disease was 0.9 years to liver decompensation in F4 [36].

## Pharmacology therapy for MASH cirrhosis

Multiple treatments that have been developed for the management of MASH cirrhosis include the following: fibroblast growth factor 21 analog, fibroblast growth factor 19 analog, galectin-3 inhibitor, pan-caspase inhibitor, apoptosis signal-regulating kinase 1 inhibitor, lysyl oxidase-like 2 monoclonal antibody, and glucagon-like peptide-1 receptor agonist. However, thus far, only some of the mentioned treatments have shown promising results regarding the primary endpoint. The Aldafermin phase 2 b trial demonstrated favorable results of a least-squares mean difference of −0.5 (95% CI, −0.7 to −0.2) in the change in ELF after 48 weeks of treatment compared to placebo [23]. Similarly, Efruxifermin trial showed ELF score improvement (−0.4 Efruxifermin vs +0.4 placebo; *P *=* *0.0036) and one or more stage improvement in fibrosis without worsening of MASH in the treatment group (33% vs 0%) [18]. Regarding HVPG, the Belapectin trial showed a significant least-square mean (SD) CFB when it was given at a dose of 2 mg/kg compared to placebo (−1.61 [0.66] vs 0.40 [0.57], respectively) [15]. Conversely, the Semaglutide phase 2 trial showed no significant improvement in 1 or more stage improvement in fibrosis without worsening of MASH (OR 0.28, 95% CI 0.06–1.24) [22]. The Simtuzumab phase 2 b trial showed no significant difference in mean difference in HVPG between the treatment and placebo group (0.1 mmHg, 95% CI −1.2 to 1.4) [20]. The Selonsertib trial showed no difference between the percentage of those with one or more stage improvement in fibrosis without worsening of MASH in the treatment group (14%) compared to the placebo group (13%) [19]. The Emricasan trial had a small treatment effect on HVPG (−0.45 mmHg vs −0.58 mmHg, treatment vs placebo, respectively), but its results were not statistically significant [17]. Lastly, the Pegbelfermin trial (FALCON 2) did not meet its primary endpoint of 1 or more stage improvement in fibrosis without worsening of MASH [14].

There have also been significant efforts made in developing pharmacological therapies for MASH cirrhosis. Resmetirom, which is an FDA-approved therapy for MASLD/MASH, is recruiting to evaluate the effect in patients with well-compensated MASH cirrhosis known as the MAESTRO-MASH-OUTCOMES (NCT05500222) [37]. Pegozafermin phase 3 study is recruited to evaluate the efficacy and safety in subjects with compensated cirrhosis due to MASH, ENLIGHTEN-Cirrhosis (NCT06419374) [38]. Efinopegdutide phase 2a study is recruiting to evaluate the efficacy and safety in adults with compensated cirrhosis secondary to MASH, MK-6024–017 (NCT06465186) [39].

## Discussion

This review summarizes the current evidence of liver cirrhosis in MASH and shines a unique aspect of the contemporary understanding of the disease. Concerning other liver diseases, liver cirrhosis is unique in MASH, where renal dysfunction and ascites are among the commonest complications of MASH cirrhosis. The field of MASH has moved significantly in the last decade, with the FDA’s first approved treatment for non-cirrhosis MASH earlier this year. Recent results from Novo Nordisk ESSENCE (Semaglutide) study presented at The Liver Meeting 2024 also show promising evidence where at week 72, 62.9% of people treated with Semaglutide 2.4 mg achieved resolution of steatohepatitis without worsening of liver fibrosis, and 37.0% achieved improvements in liver fibrosis without worsening of steatohepatitis. Additionally, the Pegozafermin phase 2b trial (ENLIVEN) has demonstrated robust fibrosis improvement at 24 weeks with 1 or more stage fibrosis improvement in 82% and 1 or more stage fibrosis improvement without worsening of MASH in 45% [40]. However, MASH cirrhosis remains an enigma, with many failures in the development of the therapeutic space potentiated by our lack of understanding of the disease. The natural history of MASH cirrhosis remains complex. Still, the evidence summarized in this review from the placebo arms of the clinical trials allows a glimpse into the natural history of the disease. Clinical trials remain the only measure of 2-point histology data accessible in MASH cirrhosis which would be nearly impossible to be done in routine clinical care. In our review, histology markers for MASH were resolution of MASH, ≥1 stage improvement in fibrosis, and ≥2-point improvement in NAS, which occurred in an average of 5%, 16%, and 32%, respectively. These changes occurred across relatively short follow-ups, where, on average, trials lasted between 26 to 96 weeks, with the majority ending around 48 weeks.

The presumed reason for this variability in results may be the possibility of reversal of cirrhosis or errors that might occur during sampling or interpreting. Liver cirrhosis is primarily understood as a one-way disease, where reversal is traditionally considered impossible. However, experimental studies have shown evidence of the regression in animal models involving the production or degradation of extracellular matrix by matrix metalloproteinases in the setting of decreased or increased tissue inhibitor of metalloproteinases-1, respectively [41]. Although it is hard to extrapolate such findings into human subjects, some truth remains about the reversal of liver fibrosis in cirrhosis. Additionally, another possibility is caused by interpretation errors. The normal liver span is usually <16 cm, ranging between 13–14 cm, and biopsy often provides 1–2 cm in length with a diameter of 1.2–1.8 mm, which is only a small fraction of the liver. Studies have also examined the possibility of sampling errors and intra-observer variation in evaluating MASH. A study by Davison *et al.* [42] report that about 16% of patients were determined to have “met” the primary endpoint when the same biopsy was re-read a second time. Additionally, Regev *et al.* [43] report that 24.2% had a difference of at least one grade in inflammation, and 33.1% had a difference of at least one stage in fibrosis between the right and left lobe. While this was done in non-MASH cirrhosis subjects, it remains reasonable to extrapolate the conclusions of the Regev *et al.* [43] study to the current review.

Considering the possible deficiencies of biopsy in detecting the regression of fibrosis, non-invasive fibrosis markers have shown promising results. In a study by Sanyal *et al.* [44], the Area Under the Receiver Operating Characteristic curves (AUROCs) for the diagnosis of cirrhosis were 0.81 for fibrosis-4 (FIB-4), 0.855 for ELF (*P *<* *0.001 versus FIB-4), and 0.897 for VCTE (*P *=* *0.002 versus FIB-4), which has been adequate in terms of diagnosis. Similarly, to monitor the regression of fibrosis, a study by Sanyal *et al.* [45] which involved 1,135 MASH patients with cirrhosis reported that those with cirrhosis regression had a significant decrease in ELF test and liver stiffness measurement by VCTE over time. Likewise, most RCTs include both histological outcomes, as well as fibrosis markers outcomes to detect regression which conclusively are the finest modality to follow regression.

This review highlights the unique aspects of liver cirrhosis in MASH and underscores the complexities and challenges in understanding the expected clinical scenarios in this group of patients. Ultimately, while strides have been made in addressing MASH and its related fibrosis, MASH cirrhosis remains an enigmatic and evolving frontier. Continued efforts to refine histological assessments, expand non-invasive diagnostic modalities, and deepen our understanding of the disease’s pathophysiology are essential to unlocking more effective therapeutic strategies.

## Authors’ contributions

D.K., C.H.N., and M.D.M. conceived and designed the project. D.K. and P.D. collected the data. M.N., C.R., and K.W. analyzed and interpreted the data. D.K., D.H.K., and P.D. drafted the manuscript. C.H.N., K.W., and M.D.M. reviewed ensuring consistency and clarity in the manuscript. All authors approve the final version of the manuscript and agree to be accountable for all aspects of the work to ensure that questions related to the accuracy or integrity of any part of the work are appropriately investigated and resolved.

## Supplementary Material

goaf037_Supplementary_Data
