# Supplementary material for: Liver cirrhosis in metabolic dysfunction-associated steatohepatitis
Source: Gastroenterol Rep (Oxf). 2025 May 2;13:goaf037. doi: 10.1093/gastro/goaf037 (PMC12065432; doi:10.1093/gastro/goaf037)
Supplement: goaf037_Supplementary_Data [file goaf037_supplementary_data.zip › Supplementary Table 1 reviewed.docx]

| Supplementary Table 1. Characteristics of included randomized clinical trials of MASH cirrhosis | | | | | | | |
| --- | --- | --- | --- | --- | --- | --- | --- |
|  | **PMID** | **Year** | D**iagnostic modality** | **Placebo (n)** | **Women (%)** | **Intervention** | **Duration (weeks)** |
| Harrison et al. | 36644237 | 2023 | Biopsy | 5 | 30 | efruxifermin 50mg | 26 |
| Garcia-Tsao et al. | 31870950 | 2020 | Biopsy | 55 | 67.2 | emricasan 50mg | 48 |
| Loomba et al. | 36934740 | 2023 | Biopsy | 24 | 75 | semaglutide 2.4mg | 48 |
| Harrison et al. | 32147362 | 2020 | Biopsy | 172 | 59 | selonsertib 18mg | 48 |
| Frenette et al. | 33038432 | 2021 | Biopsy | 70 | N/A | emricasan 25mg | 48 |
| Loomba et al. | 33169409 | 2020 | Biopsy | 21 | 69 | firsocostat/ cilofexor | 48 |
| Rinella et al. | 37732990 | 2024 | Biopsy | 56 | 69.6 | aldafermin 3mg | 48 |
| Abdelmalek et al. | 37088458 | 2024 | Biopsy | 39 | 61.5 | pegbelfermin 40mg | 48 |
| Chalasani et al. | 31812510 | 2020 | Biopsy | 54 | 67 | belapectin 8mg/kg | 52 |
| Harrison et al. | 29990488 | 2018 | Biopsy | 85 | 66 | simtuzumab 700mg | 96 |
